# Studying eruption patterns of mandibular third molars for forensic age assessment: Introducing current reference data from a population of black South Africans

**DOI:** 10.1007/s00414-024-03251-x

**Published:** 2024-05-11

**Authors:** Maximilian Timme, Jan Viktorov, Laurin Steffens, Adam Streeter, André Karch, Chané Smit, Liam Robinson, Herman Bernitz, Andreas Schmeling

**Affiliations:** 1 Institute of Legal Medicine, University Hospital Münster, Röntgenstraße 23, 48149 Münster, Germany; 2 Institute of Epidemiology and Social Medicine, University of Münster, Domagkstraße 3, 48149 Münster, Germany; 3 Department of Oral and Maxillofacial Pathology, Faculty of Health Sciences, University of Pretoria, Gauteng, South Africa

**Keywords:** Age estimation, Dental age, Wisdom tooth, Dental panoramic radiograph, Chronological age

## Abstract

**Introduction:**

Evaluation of the eruption of mandibular third molars in orthopantomograms (OPGs) is a method of forensic age assessment. The objective of our study was to provide valid reference data for this trait within a population of black South Africans. The study was guided by the criteria for reference studies in age assessment.

**Materials and methods:**

A study population from Pretoria, South Africa comprising 670 OPGs obtained from 338 black females and 332 black males aged between 15.00 and 25.97 years was analysed. All OPGs were performed for medical indication during the period from 2011 to 2022 and were retrospectively evaluated. From the 670 OPGs, a total of 1021 mandibular third molars were evaluated. The assessment of mandibular third molars was conducted using the staging scale presented by Olze et al. in 2012. Two experienced dentists evaluated the OPGs independently of each other. If the two examiners diverged in their assessments, a consensus stage was assigned.

**Results:**

As expected, the mean, median and minimal age increased with higher stages for both teeth and both sexes. The minimum age recorded for stage D, indicating complete tooth eruption, was 15.79 years in females and 16.62 years in males.

**Conclusion:**

As it is the case for previous reference studies in other countries, placing exclusive reliance on the evaluation of mandibular third molar eruption is inadequate for ascertaining the age of majority among Black South Africans. Future studies need to examine if our results are transferable to other countries in Sub-Saharan Africa.

## Introduction

Scientific forensic age assessment in the living is typically used whenever a person’s age is unknown or if there are reasonable doubts about it [1]. It is thought to support legal processes in various areas of law [1], for example, protecting the status of unaccompanied refugee minors [2–5]. This legal issue is increasingly important due to the recent sharp rise in cross-border migration movements [6].

The field of forensic age assessment in the living currently accounts for a considerable proportion of research activities in forensic science [7–17]. The focus of scientific efforts in the field today is the establishment of new methods or the further development of established evidence-based methods.

As there are no recent conclusive guidelines on implementing age assessment procedures for living individuals, the international and interdisciplinary Study Group of Forensic Age Diagnostics (AGFAD) recommendations from 2008 are still considered the gold standard [18]. These recommendations specify the scope of examinations that every forensic age assessment should include. Thus, a physical examination with determination of anthropometric measures, inspection of signs of sexual maturation, and identification of any age-relevant developmental disorders is required. If the skeletal development of the hand is completed, an additional radiographic examination of the clavicles should be carried out [18].

The recommendations also call for a dental examination, including a radiographic examination of the dentition [18]. Dental panoramic radiography or orthopantomography (orthopantomogram - OPG) has become the preferred radiographic examination technique [1]. The advantages of OPGs lie in its ability to assess the entire jaw and dentition, ease of availability, low costs, and the ratio of radiation exposure to information value [19]. Since a large number of cases of forensic age assessment is concerned explicitly with the question of age majority (herewith referred to as the completion of the 18th year of life) of the person being examined, tooth development characteristics that typically have not been completed at this age must be examined accordingly. The third molars are usually the teeth that develop last [20], completing mineralization at around the age of 18 years or a few years beyond [20]. For this reason, the assessment of eruption and mineralization of the third molars has become established in forensic age assessment practice [1].

There is currently a lack of comparable, systematic studies of third molar eruption as a feature of forensic age assessment in different ethnic groups. The current study aimed to provide reference data on the eruption of mandibular third molars using OPGs from a study population of black South Africans.

## Materials and methods

The study received positive ethics votes from both the University of Pretoria (No. 587/2021), the origin of the data, and the University of Münster (No. 2020-038-f-S), the venue of the study.

The orthopantomograms (OPGs) utilized in this investigation were sourced from the University of Pretoria Oral Health Centre, Pretoria, Gauteng, South Africa. All OPGs employed were initially obtained for medico-dental diagnostic purposes. In this study, OPGs were randomly selected from all available OPGs for retrospective, blinded assessment, with stratification based on each year of age within the 15-25-year range, aligning with established practices in comparable literature [21]. Individuals were categorized according to their current year of age, e.g., ages between 15.00 and 15.99 years were grouped as “15 years”. Participants were only included in the study if their age at the time of radiographic examination was unequivocally known. Upon admission to the dental clinic, patients under the age of 16 must present a state birth certificate. Persons older than 16 years of age receive an identification card in South Africa, which is then required for admission to the dental clinic.

The study was carried out on self- classified black individuals. The socioeconomic status of South Africa as a whole is described as an upper-middle-income region [22]. However, this classification, which is based on gross national income (GNI), is a distorted reflection of the reality of life for a large number of South Africans. This is characterized by poverty in large parts of the population. In particular, the socioeconomic background of black people in South Africa is still worse than that of the average population [23]. In addition, the public healthcare institutions in South Africa tend to be utilized by people of lower socioeconomic status [24]. Therefore, the overall socioeconomic status of the study population is below the average for South Africa and certainly cannot be regarded as an upper-middle-income population.

The initial phase of this study involved the collection of OPG images, guided by criteria encompassing suitable age, sex, and the radiographic visibility of at least one mandibular third molar. Subsequently, exclusion criteria specific to individual images were applied during evaluations.

The first step was to check that the image quality was sufficient. The exclusion criteria here comprised issues related to artifacts or misalignments during image acquisition, resulting in distortions. In the second step, images with pathologies were sorted. For example, bone fractures, cysts, carious lesions on the third molars, dental restorations on the third molars, or orthodontic appliances on the third molars were considered excluded. In addition, third molars with an angulation of more than 30 degrees in the mesio-distal direction were excluded, as a physiological eruption cannot be assumed in these instances [25, 26]. Only one OPG from the same individual was considered for inclusion.

The evaluations were performed according to the classification *by Olze et al. (2012)* [27]:

*A* coverage of the occlusal surface with alveolar bone.

*B* alveolar emerge, complete resorption of the alveolar bone over the occlusal surface.

*C* at least half the crown length of the second molar has been reached, the occlusal plane has not been reached.

*D* complete emerge in the occlusal plane.

Radiographs were scrutinized employing Synedra Personal View software version 22.0.0 (Synedra Information Technologies GmbH, Innsbruck, Austria) on designated workstations. The configuration and environmental conditions remained stable for both examiners. The software’s magnification and grey-level adjustment tools were deployed throughout the assessments. The examiners, two board-certified dentists, possessed extensive familiarity with the methodology gleaned from previous studies [28]. Both examiners conducted Independent evaluations of the radiographs. In cases where discrepant determinations arose, a consensus was reached through subsequent deliberation between the two examiners.

The mean, median, upper and lower quartile, and minimum and maximum age were determined for each stage, stratified by sex and tooth. Data management and statistical analyses were executed using Stata, version 13.0 (Stata Corp LP, College Station, Texas, USA).

## Results

The study included a total of 670 orthopantomograms (OPGs), comprising 338 females and 332 males, spanning ages from 15.00 (female) to 25.97 (female) years. Image acquisition occurred between December 2011 and December 2022. Table 1 provides an overview of the study population stratified by age and biological sex.

**Table 1:** Age and sex distribution of the study population

Age	Males (*n*)	Females (*n*)	Total (*n*)
15	26	24	50
16	23	25	48
17	29	24	53
18	29	35	64
19	30	30	60
20	30	37	67
21	26	33	59
22	31	30	61
23	32	35	67
24	33	31	64
25	43	34	77
Total (n)	332	338	670

Considering the total collected radiographs, an analysis of 1340 teeth was potentially feasible. However, due to the absence of some mandibular third molars and the stringent application of exclusion criteria, a total of 1021 teeth (males: 491, females: 530) met the inclusion criteria (Table 2). These 1021 teeth constitute the foundation for the reference data. The number of examined teeth, which form the actual basis of the reference data, varied from *n* = 19 (tooth 48 [FDI system], age group 16 years, males) to *n* = 31 (tooth 38 [FDI system], age group 20 years, females) (Table 2).

**Table 2:** Number of teeth evaluated by age and sex

Age	Tooth [FDI]	Males (*n*)	Females (*n*)	Total (*n*)
15	38	25	23	48
	48	25	23	48
16	38	21	23	44
	48	19	24	43
17	38	22	22	44
	48	23	18	41
18	38	21	27	48
	48	15	24	39
19	38	21	22	43
	48	21	24	45
20	38	21	31	52
	48	23	28	51
21	38	22	27	49
	48	23	28	51
22	38	25	23	48
	48	20	22	42
23	38	25	21	46
	48	23	23	46
24	38	21	26	47
	48	24	23	47
25	38	29	22	51
	48	22	26	48
Total (n)		491	530	1021

As expected, the mean, median, and minimum ages at each stage increased with stages for both sexes and both teeth (Tables 3, 4, 5 and 6). The minimum age at stage D for females for tooth 38 was found to be 15.79 years. For tooth 48, this value was 16.87 years. The minimum age at stage D was 16.62 years for males in both examined teeth. Hence, the recorded minimum ages associated with stage D, indicative of full eruption, were considerably below the threshold of 18 years for both teeth and both sexes.

**Table 3:** Descriptive measures for each stage for tooth 38 [FDI] in females. Age in years, rounded to second decimal place. SD: standard deviation. LQ: lower quartile. UQ: upper quartile

Stage	*N*	Mean	SD	Median	LQ	UQ	Min	Max
A	43	16.73	1.91	16.14	15.48	17.31	15.00	25.24
B	23	18.23	2.34	17.72	16.31	20.48	15.16	22.48
C	45	20.27	2.85	19.66	18.29	22.95	15.20	25.88
D	156	21.89	2.38	21.97	20.17	24.03	15.79	25.92

**Table 4:** Descriptive measures for each stage for tooth 48 [FDI] in females. Age in years, rounded to second decimal place. SD: standard deviation. LQ: lower quartile. UQ: upper quartile

Stage	*N*	Mean	SD	Median	LQ	UQ	Min	Max
A	41	16.75	1.90	16.13	15.36	17.31	15.00	23.23
B	24	18.42	2.75	18.23	16.23	19.70	15.04	25.88
C	43	20.56	2.83	19.91	18.38	23.17	15.79	25.66
D	155	21.97	2.46	21.98	20.11	24.16	16.87	25.97

**Table 5:** Descriptive measures for each stage for tooth 38 [FDI] in males. Age in years, rounded to second decimal place. SD: standard deviation. LQ: lower quartile. UQ: upper quartile

Stage	*N*	Mean	SD	Median	LQ	UQ	Min	Max
A	37	17.65	3.00	16.11	15.58	19.46	15.03	25.20
B	47	18.37	2.72	17.53	16.13	19.38	15.12	25.34
C	36	21.01	3.07	21.42	18.27	23.38	15.39	25.93
D	133	22.11	2.50	22.52	20.31	24.22	16.62	25.93

**Table 6:** Descriptive measures for each stage for tooth 48 [FDI] in males. Age in years, rounded to second decimal place. SD: standard deviation. LQ: lower quartile. UQ: upper quartile

Stage	*N*	Mean	SD	Median	LQ	UQ	Min	Max
A	41	17.73	3.00	16.28	15.58	18.46	15.03	25.22
B	40	18.43	2.65	17.51	16.42	19.39	15.12	24.82
C	31	21.06	3.12	21.40	19.01	23.90	15.14	25.68
D	126	21.97	2.49	22.12	20.31	24.15	16.62	25.93

The medians from stage C onwards were above 18 years for both sexes and both teeth. Only for tooth 48 in females was the median already above this age threshold in stage B. The values for the lower quartile for stage C were above 18 years for both sexes and both teeth.

Stage D covered almost the entire age range of the study population for both teeth and both sexes. This was most evident for tooth 38 in females, where the minimum age for stage D was 15.79 years. For all other teeth and sexes examined, the minimum age for stage D was over 16 years.

The prevalences of the different stages varied, ranging from 23 cases (stage B, tooth 38, females) to 156 cases (stage D, tooth 38, females), with stage D being the most frequently observed stage across both teeth and both sexes (Tables 3, 4, 5 and 6).

## Discussion

In this study, we examined the age-specific third molar eruption patterns of Black South Africans using a standardized assessment based on the most recent recommendations for age assessment studies. This ethnicity-specific approach was chosen because the question of the dependence of the temporal patterns of third molar eruption on ethnicity had recently been raised in the literature [29]. Specifically, the assumption was made that Africans pass through the stages of third molar eruption faster than Asians or Europeans [29, 30]. It should be pointed out that the presentation of the reference data for the population in focus means that a direct comparison between different populations is only possible to a very limited extent. Reliable reference data from different ethnic groups are essential in order to be able to investigate this question scientifically in the future.

The temporal patterns of tooth eruption in African populations have been widely studied. In a 2021 review, Cummaudo et al. were able to compile 25 studies on this topic alone [31]. However, the methodology and definition of ethnicity were inconsistent. Additionally, forensic age estimation was usually not the focus of these studies, which was why the eruption of third molars, in particular, was not assessed in a standardized way [31].

Few studies are available examining the temporal patterns of mandibular third molar eruption in Black Africans for forensic age estimation. Kutesa et al. and Olze et al. conducted studies on Black Africans using the staging scale by Olze et al. from 2007 [29, 32, 33]. These two studies are directly compared to the current study in Table 7.

**Table 7:** Comparison of the studies investigating eruption of mandibular third molars using the Olze et al. method in black African populations and the present study. For better comparability, the values of the present study are included again, although they are already listed in Tables 3–6. SD: standard deviation. LQ: lower quartile. UQ: upper quartile. - : No information given. Values for stage C in *italics* because the staging scale for stage C in the present study is not the same. Values are rounded to one decimal place

	Kutesa et al. 2019 [33]	Olze et al. 2007 [32]	Present study
General characteristics			
Individuals (n) |Teeth included (n) Total	1012 | -	516 | -	670 | 1021
Females	541 | -	106 | -	338 | 530
Males	471 | -	410 | -	332 | 491
Age groups [years]	10–20	12–26	15–25
Reliability of participants’ age information	Low	High	High
Geographic origin of the study population	Uganda (Kampala)	South Africa (Pretoria)	South Africa (Pretoria)
Ethnicity	Black	Black	Black
Study design	retrospective	retrospective	retrospective
Staging scale	Olze et al. 2007 [29]	Olze et al. 2007 [29]	Olze et al. 2012 [27]
Generation period of X-rays	01/2017–12/2017	1992–2002	12/2011–12/2022
Results			
Intra-rater agreement [kappa]	0.81–0.96	0.88–0.96	Consensus
Inter-rater agreement [kappa]	0.96	-	
Descriptive measures Mean; SD | Min; Max | LQ; Median; UQ			
Stage A Sex; tooth [FDI system]			
Males; 38	12.3; 2.3 | 10; 19 | 11; 12; 13	13.6; 1.4 | 12.0; 15.7 | 12.5; 13.3; 14.9	17.7; 3.0 | 15.0; 25.2 | 15.6; 16.1; 19.5
Males; 48	12.3; 2.3 | 10; 19 | 11; 12; 13.5	13.6; 1.3 | 12.0; 15.7 | 12.6; 13.5; 14.7	17.3; 3.0 | 15.0; 25.2 | 15.6; 16.3; 18.5
Females; 38	12.0; 1.8 | 10; 18 | 11; 12; 13	15.4; 0.8 | 14.9; 16.0 | 14.9; 15.4; 16.0	16.3; 1.9 | 15.0; 25.2 | 15.5; 16.1; 17.3
Females; 48	12.0; 1.9 | 10; 19 | 10.5; 12; 13	16.0; 1.2 | 14.9; 17.2 | 14.9; 16.0; 17.2	16.8; 1.9 | 15.0; 23.2 | 15.4; 16.1; 17.3
Stage B			
Males; 38	15.7; 2.0 | 11; 19 | 14; 16; 18	15.8; 0.9 | 15.4; 26.1| 15.7; 17.8; 22.6	18.4; 2.7 | 15.1; 25.3 | 16.1; 17.3; 19.4
Males; 48	15.4; 2.1 | 11; 19 | 14; 15; 18	17.2; 1.9 | 15.1; 20.3 | 15.4; 16.4; 19.2	18.4; 2.7 | 15.1; 24.8 | 16.4; 17.5; 19.4
Females; 38	14.8; 2.2 | 10; 19 | 13.5; 15; 16	15.7; 2.7 | 12.1; 21.9 | 13.8; 15.5; 17.2	18.2; 2.3 | 15.2; 22.5 | 16.3; 17.7; 20.5
Females; 48	14.9; 2.0 | 10; 19 | 14; 15; 16	15.1; 2.0 | 12.1; 18.6 | 13.8; 14.6; 16.7	18.2; 2.8 | 15.0; 25.9 | 16.2; 18.2; 19.7
Stage C			
Males; 38	16.5; 2.1 | 12; 19 | 15; 17.5; 18	20.8; 2.8 | 18.4; 24.2 | 18.5; 20.2; 23.6	*21.0;0.3.1 | 15.4; 25.9 | 18.3; 21.4; 23.4*
Males; 48	16.8; 1.6 | 14; 19 | 15; 17; 18	20.7; 2.4 | 18.4; 24.2 | 18.6; 20.8; 23.4	*21.1; 3.1 | 15.1; 25.7 | 19.0; 21.4; 23.9*
Females; 38	15.8; 2.1 | 10; 18 | 15; 16; 18	18.6; 4.7 | 14.5; 23.8 | 14.5; 17.6; 23.8	*20.3; 2.9 | 15.2; 25.9 | 18.3; 19.7; 23.0*
Females; 48	15.2; 2.2 | 10; 18 | 13.5; 15; 17.5	19.9; 5.4 | 16.1; 23.8 | 16.1; 19.9; 23.8	*20.6; 2.8 | 15.8; 25.7 | 18.4; 19.9; 23.2*
Stage D			
Males; 38	18.2; 1.6 | 13; 20 | 17; 19; 20	22.4; 2.2 | 16.7; 26.9 | 20.9; 22.6; 24.2	22.1; 2.5 | 16.6; 25.9 | 20.3; 22.5; 24.2
Males; 48	18.2; 1.6 | 13; 20 | 17; 19; 20	22.6; 2.2 | 17.0; 26.9 | 21.1; 22.8; 24.2	22.5; 2.4 | 16.6; 25.9 | 20.3; 22.1; 24.2
Females; 38	17.5; 1.9 | 13; 20 | 16; 18; 19	21.7; 2.8 | 16.1; 26.8 | 19.8; 22.2; 23.7	21.9; 2.4 | 15.8; 25.9 | 20.2; 22.0; 24.0
Females; 48	17.6; 1.9 | 13; 20 | 16; 18; 19	22.0; 2.7 | 16.1; 26.8 | 20.2; 22.4; 23.7	22.0; 2.5 | 16.9; 26.0 | 20.1; 22.0; 24.2

The different age intervals of the studies become apparent, whereby the age ranges of the study by Olze et al. and the current study are most comparable. The study by Kutesa et al. provides data with a considerably younger age distribution, which may have influenced the final results.

For studies on forensic age assessment, an equal distribution of individuals within the age groups is recommended [18]. An uneven distribution can lead to bias in the results. In particular, the mean age in the stages is prone to bias when uneven age distributions are used [34]. The effect of uneven age distribution can be described qualitatively by stating that disproportionate proportions of younger individuals lead to lower estimated ages, and disproportionate proportions of older individuals lead to higher estimated ages [34]. However, in studies that examine tooth traits, possibly on several teeth, it is not necessarily fair to infer the number of teeth examined from the number of individuals included. This is crucial, as the teeth examined ultimately form the basis for the reference values obtained. Therefore, we have also presented the number of teeth examined in our study in Table 2 to reveal the actual basis of our reference data.

In addition to an equal distribution of examined traits, the selection of age intervals in studies is also crucial [34, 35]. If different studies are to be compared, it is decisive to account for the age intervals in the individual studies. The effect of different age intervals is qualitatively similar to the unequal distribution of age groups [34]. For example, including younger age groups will reduce the estimated ages. The same applies to the absence of older age groups. Olze et al. included relatively few young males aged 12–16 years and generally very few females in their study [32]. For example, the 16-year-old female age group only included 2 individuals, and the 12-year-old female age group only included 3 individuals. In the 13- and 14-year-old female age groups, only 4 individuals were studied in each case. The study by Kutesa et al. also did not have an exact equal distribution, with 66 test subjects in the 10-year-old female age group and only 29 individuals in the 20-year-old female age group. Overall, young females aged 10–13 years are overrepresented in Kutesa et al., while the age groups 17–20 years are overrepresented among males [33]. For our study, the included teeth can be seen directly in Table 2. There is also no exact equal distribution, although a clear trend cannot be determined. Due to the age range studied, our study cannot contribute to any statement about the behavior of the feature in people under the age of 15.

When the reference values of the three studies in Table 7 are compared, the different minimum ages in stage D, i.e., the minimum age for a fully completed eruption of the mandibular third molars, are particularly evident. Kutesa et al. mostly present values rounded to whole years, finding a minimum age of 13 years for the completed eruption [33]. These values are at least 3 years below the values found in our study. For Kutesa et al., the completed eruption at 13 years is not an isolated case; rather, they found this in 5 cases for tooth 38 and 4 cases for tooth 48 [33]. These values are remarkable, as Olze et al. found a minimum age for stage D of 16.1 years, although they included individuals aged 12 years and older in their study [32]. However, it should be noted that age groups 12, 13, and 14 years were relatively underrepresented in the study by Olze et al., and they could, therefore, have missed stage D cases. However, when analyzing the data from Kutesa et al., details of the age information control of their study participants must be considered [33]. They state that at the time of the study, only people over the age of 16 had a national identity card in Uganda, which is similar to the procedure in South Africa. However, the difference in the study by Kutesa et al. concerns people under the age of 16, as various less reliable documents such as baptismal certificates, birth certificates (unclear exactly what kind), or vaccination certificates were consulted [33]. The reliability of these documents must be called into question and thus reflects the entire problem of forensic age assessment. Age information from certain regions is not always reliable for various reasons [36], making forensic age assessment necessary [37]. Therefore, the data from the study by Kutesa et al. should be assessed with these limitations in mind.

Obviously, it must be taken into account that even official documents may not be 100% reliable.

This aspect must also be taken with regard to the present study, although South Africa was deliberately chosen as the study location, as official government documents can generally be considered reliable here.

A relevant difference between our results and those of Olze et al. is that the current study detected a stage D below the age of 16 in females in tooth 38 [32]. However, it should also be noted here that the female age group of 15 years in the study by Olze et al. consisted of only 5 individuals, probably resulting in missed stage D cases.

The existing literature on age assessment often uses inconsistent methods or staging scales for the same trait [16, 28, 38, 39]. The choice of staging scale often falls on the most recent or widely used system [40]. For our study, we chose a different approach. Our study was preceded by a preliminary study in which we investigated the question of the most suitable staging scale for assessing mandibular third molars on OPGs [28]. We compared different staging scales and clarified the question of the best correlation with age and the best reliability. This preliminary study showed that the staging scale by Olze et al. from 2012 is particularly appropriate and should be used in further studies [28]. For this reason, we used this staging scale in the current study. The staging scale by Olze et al. from 2012 is a further development of an older version from 2007. The two versions of the staging scale differ only in the definition of stage C [27, 39, 41]. Nevertheless, the comparability of the study results in Table 7 is somewhat limited.

Another distinctive feature of the current study concerns the consensual observer agreement. In studies on forensic age assessment, it is often common practice to determine the observer agreement between the different examiners and to indicate it utilizing kappa coefficients [42–44]. However, if this principle is applied in reference studies, there is a risk that “incorrect” assessments by the main examiner will be included in the reference data. Therefore, we chose the consensual evaluation of the two examiners for our study to reduce the proportion of “incorrect” values in the reference data. We applied this procedure because, in our preliminary study, we also examined the observer agreements for the staging scale by Olze et al. from 2012 [28]. We found that this method achieved the highest point estimates [28]. We recommend the implementation of a consensual determination of several examiners for further reference studies on forensic age assessment.

## Conclusion

Contrary to older literature, in this study on a Black South African population, the eruption of mandibular third molars was completed before the age of 16 in females. Overall, the trait of eruption of the mandibular third molars in this population is unsuitable for detecting the completion of the 18th year of life. Future studies should examine the extent to which our results represent different populations in Sub-Saharan Africa.

## Data Availability

The datasets generated during the current study are available from the corresponding author upon reasonable request.

## References

[1] Schmeling A, Dettmeyer R, Rudolf E, Vieth V, Geserick G (2016) Forensic age estimation: methods, certainty, and the Law. Deutsches Ärzteblatt International. 10.3238/arztebl.2016.0044 26883413 10.3238/arztebl.2016.0044 PMC4760148

[2] Schmeling A, Geserick G, Tsokos M, Dettmeyer R, Rudolf E, Püschel K (2014) Aktuelle Diskussionen Zur Altersdiagnostik bei unbegleiteten minderjährigen Flüchtlingen. Rechtsmedizin 24:475–479. 10.1007/s00194-014-0986-x 10.1007/s00194-014-0986-x

[3] Nuzzolese E, Di Vella G (2008) Forensic dental investigations and age assessment of asylum seekers. Int Dent J 58:122–126. 10.1111/j.1875-595x.2008.tb00186.x 18630106 10.1111/j.1875-595x.2008.tb00186.x

[4] Olze A, Reisinger W, Geserick G, Schmeling A (2006) Age estimation of unaccompanied minors. Part II. Dental aspects. Forensic Sci Int 159(Suppl 1):S65–67. 10.1016/j.forsciint.2006.02.018 16529893 10.1016/j.forsciint.2006.02.018

[5] Schmeling A, Reisinger W, Geserick G, Olze A (2006) Age estimation of unaccompanied minors. Part I. General considerations. Forensic Sci Int 159(Suppl 1):S61–64. 10.1016/j.forsciint.2006.02.017 16529895 10.1016/j.forsciint.2006.02.017

[6] McAuliffe M, Triandafyllidou A (eds) (2021) World Migration Report 2022. International Organization for Migration (IOM), Geneva

[7] Cameriere R, Scendoni R, Ferrante L, Mirtella D, Oncini L, Cingolani M (2023) An effective model for estimating age in unaccompanied minors under the Italian legal system. Healthc (Basel) 11:224. 10.3390/healthcare11020224 10.3390/healthcare11020224 PMC9859195 36673592

[8] Wang C, Tian Z, Wen D, Qu W, Xu R, Liu Y, Jia H, Tang X, Li J, Zha L, Liu Y (2023) Preliminary study on genetic factors related to Demirjian’s tooth age estimation method based on genome-wide association analysis. Int J Legal Med 137:1161–1179. 10.1007/s00414-023-03008-y 37133749 10.1007/s00414-023-03008-y

[9] Wang J, Zhang H, Wang C, Fu L, Wang Q, Li S, Cong B (2022) Forensic age estimation from human blood using age-related microRNAs and circular RNAs markers. Front Genet 13:1031806. 10.3389/fgene.2022.1031806 36506317 10.3389/fgene.2022.1031806 PMC9732945

[10] Bjelopavlovic M, Reder SR, Fritzen I, Brockmann MA, Hardt J, Petrowski K (2023) Forensic age estimation: a Multifactorial Approach in a Retrospective Population Study. Diagnostics (Basel) 132029. 10.3390/diagnostics13122029 10.3390/diagnostics13122029 PMC10297483 37370924

[11] Yavuz TK, Hilal A, Kaya O, Ekizoglu O, Kaya K (2023) Forensic age estimation with ankle MRI: evaluating distal tibial and calcaneal epiphyseal fusion. Forensic Sci Int 352:111832. 10.1016/j.forsciint.2023.111832 37776598 10.1016/j.forsciint.2023.111832

[12] Luo S, Fan F, Zhang XT, Liu AJ, Lin YS, Cheng ZQ, Song CX, Wang JJ, Deng ZH, Zhan MJ (2023) Forensic age estimation in adults by pubic bone mineral density using multidetector computed tomography. Int J Legal Med 137:1527–1533. 10.1007/s00414-023-03067-1 37493764 10.1007/s00414-023-03067-1

[13] Widek T, De Tobel J, Ehammer T, Genet P (2023) Forensic age estimation in males by MRI based on the medial epiphysis of the clavicle. Int J Legal Med 137:679–689. 10.1007/s00414-022-02924-9 36534129 10.1007/s00414-022-02924-9 PMC10085911

[14] Ruder TD, Kuhnen SC, Zech W-D, Klaus JB, Lombardo P, Ith M (2023) Standards of practice in forensic age estimation with CT of the medial clavicular epiphysis-a systematic review. Int J Legal Med. 10.1007/s00414-023-03061-7 37691040 10.1007/s00414-023-03061-7 PMC10567934

[15] Onofri M, Delicati A, Marcante B, Carlini L, Alessandrini F, Tozzo P, Carnevali E (2023) Forensic age estimation through a DNA methylation-based Age Prediction Model in the Italian Population: a pilot study. Int J Mol Sci 24:5381. 10.3390/ijms24065381 36982454 10.3390/ijms24065381 PMC10049185

[16] Timme M, Bender J, Steffens L, Shay D, Schmeling A (2023) Third molar eruption in Dental panoramic radiographs as a feature for forensic Age Assessment-Presentation of a New Non-staging Method based on measurements. Biology (Basel) 12:1403. 10.3390/biology12111403 37998002 10.3390/biology12111403 PMC10669860

[17] Vila-Blanco N, Varas-Quintana P, Tomás I, Carreira MJ (2023) A systematic overview of dental methods for age assessment in living individuals: from traditional to artificial intelligence-based approaches. Int J Legal Med 137:1117–1146. 10.1007/s00414-023-02960-z 37055627 10.1007/s00414-023-02960-z PMC10247592

[18] Schmeling A, Grundmann C, Fuhrmann A, Kaatsch H-J, Knell B, Ramsthaler F, Reisinger W, Riepert T, Ritz-Timme S, Rösing FW, Rötzscher K, Geserick G (2008) Criteria for age estimation in living individuals. Int J Legal Med 122:457–460. 10.1007/s00414-008-0254-2 18548266 10.1007/s00414-008-0254-2

[19] Masthoff M, Gerwing M, Masthoff M, Timme M, Kleinheinz J, Berninger M, Heindel W, Wildgruber M, Schülke C (2018) Dental Imaging - A basic guide for the radiologist. Rofo. 10.1055/a-0636-4129 29913523 10.1055/a-0636-4129

[20] AlQahtani SJ, Hector MP, Liversidge HM (2010) Brief communication: the London atlas of human tooth development and eruption. Am J Phys Anthropol 142:481–490. 10.1002/ajpa.21258 20310064 10.1002/ajpa.21258

[21] Olze A, Peschke C, Schulz R, Schmeling A (2008) Studies of the chronological course of wisdom tooth eruption in a German population. J Forensic Leg Med 15:426–429. 10.1016/j.jflm.2008.02.008 18761308 10.1016/j.jflm.2008.02.008

[22] The World Bank (2023), Dec 21 World Bank Country and Lending Groups. datahelpdeskworldbank.org. https://datahelpdesk.worldbank.org/knowledgebase/articles/906519-world-bank-country-and-lending-groups

[23] Gradin C (2013) Race, poverty and deprivation in South Africa. J Afr Econ 22:187–238. 10.1093/jae/ejs019 10.1093/jae/ejs019

[24] Coovadia H, Jewkes R, Barron P, Sanders D, McIntyre D (2009) The health and health system of South Africa: historical roots of current public health challenges. Lancet 374:817–834. 10.1016/S0140-6736(09)60951-X 19709728 10.1016/S0140-6736(09)60951-X

[25] Wolf H, Haunfelder D (1960) Zahnärztliche Mundchirurgie für Studierende der Zahnheilkunde. Berlinische Verlagsanstalt. Berlin. Germany

[26] Archer WH (1955) Die Chirurgie des Mundes und der Zähne. Medica. Stuttgart. Germany

[27] Olze A, Peschke C, Schulz R, Schmeling A (2012) [Application of a modified stage classification in evaluating wisdom tooth eruption in a German population]. Arch Kriminol 229:145–153 22834358

[28] Timme M, Viktorov J, Steffens L, Streeter A, Karch A, Schmeling A (2023) Third molar eruption in orthopantomograms as a feature for forensic age assessment-a comparison study of different classification systems. Int J Legal Med 137:765–772. 10.1007/s00414-023-02982-7 36884067 10.1007/s00414-023-02982-7 PMC10085935

[29] Olze A, van Niekerk P, Ishikawa T, Zhu BL, Schulz R, Maeda H, Schmeling A (2007) Comparative study on the effect of ethnicity on wisdom tooth eruption. Int J Legal Med 121:445–448. 10.1007/s00414-007-0171-9 17453230 10.1007/s00414-007-0171-9

[30] Schmeling A, Olze A, Reisinger W, Geserick G (2001) Der Einfluss Der Ethnie auf die bei strafrechtlichen Altersschätzungen Untersuchten Merkmale. Rechtsmedizin 11:78–81. 10.1007/s001940100098 10.1007/s001940100098

[31] Cummaudo M, De Angelis D, Magli F, Minà G, Merelli V, Cattaneo C (2021) Age estimation in the living: a scoping review of population data for skeletal and dental methods. Forensic Sci Int 320:110689. 10.1016/j.forsciint.2021.110689 33561788 10.1016/j.forsciint.2021.110689

[32] Olze A, van Niekerk P, Schulz R, Schmeling A (2007) Studies of the chronological course of wisdom tooth eruption in a black African population. J Forensic Sci 52:1161–1163. 10.1111/j.1556-4029.2007.00534.x 17767660 10.1111/j.1556-4029.2007.00534.x

[33] Kutesa AM, Rwenyonyi CM, Mwesigwa CL, Muhammad M, Nabaggala GS, Kalyango J (2019) Dental age estimation using radiographic assessment of third molar eruption among 10-20-year-old Ugandan population. J Forensic Dent Sci 11:16–21. 10.4103/jfo.jfds_34_19 31680751 10.4103/jfo.jfds_34_19 PMC6822306

[34] Gelbrich B, Lessig R, Lehmann M, Dannhauer K-H, Gelbrich G (2010) Altersselektion in Referenzstichproben: Auswirkung auf die forensische Altersschätzung. Rechtsmedizin 20:459–463. 10.1007/s00194-010-0703-3 10.1007/s00194-010-0703-3

[35] Knell B, Ruhstaller P, Prieels F, Schmeling A (2009) Dental age diagnostics by means of radiographical evaluation of the growth stages of lower wisdom teeth. Int J Legal Med 123:465–469. 10.1007/s00414-009-0330-2 19241087 10.1007/s00414-009-0330-2

[36] Kihara EN, Karanja SM, Wanzala P, Wagaiyu EG (2023) Age assessment services in public dental health facilities in Kenya: burden and sources of referral (a cross-sectional study). Pan Afr Med J 45:125. 10.11604/pamj.2023.45.125.37365 37790153 10.11604/pamj.2023.45.125.37365 PMC10543910

[37] Sauer PJJ, Nicholson A, Neubauer D, Advocacy and Ethics Group of the European Academy of Paediatrics (2016) Age determination in asylum seekers: physicians should not be implicated. Eur J Pediatr 175:299–303. 10.1007/s00431-015-2628-z 26385241 10.1007/s00431-015-2628-z

[38] Gambier A, Rérolle C, Faisant M, Lemarchand J, Paré A, Saint-Martin P (2019) Contribution of third molar eruption to the estimation of the forensic age of living individuals. Int J Legal Med 133:625–632. 10.1007/s00414-018-01991-1 30635722 10.1007/s00414-018-01991-1

[39] Olze A, Peschke C, Schulz R, Ribbecke S, Schmeling A (2012) Beurteilung Der Weisheitszahneruption: Vergleich Von Zwei Stadieneinteilungen. Rechtsmedizin 22:451–455. 10.1007/s00194-012-0845-6 10.1007/s00194-012-0845-6

[40] Švábová Nee Uhrová P, Beňuš R, Chovancová Nee Kondeková M, Vojtušová A, Novotný M, Thurzo A (2023) Use of third molar eruption based on Gambier’s criteria in assessing dental age. Int J Legal Med 137:691–699. 10.1007/s00414-023-02953-y 36707450 10.1007/s00414-023-02953-y

[41] Olze A, Bilang D, Schmidt S, Wernecke K-D, Geserick G, Schmeling A (2005) Validation of common classification systems for assessing the mineralization of third molars. Int J Legal Med 119:22–26. 10.1007/s00414-004-0489-5 15538611 10.1007/s00414-004-0489-5

[42] Franceschetti L, Merelli VG, Corona S, Magli F, Maggioni L, Cummaudo M, Tritella S, De Angelis D, Cattaneo C (2022) Analysis of interrater reliability in age assessment of minors: how does expertise influence the evaluation? Int J Legal Med 136:279–285. 10.1007/s00414-021-02707-8 34591185 10.1007/s00414-021-02707-8 PMC8813704

[43] Chaudhary MA, Liversidge HM (2017) A radiographic study estimating age of mandibular third molars by periodontal ligament visibility. J Forensic Odontostomatol 35:79–89 29384739 PMC6100229

[44] Koranne VV, Mhapuskar AA, Marathe SP, Joshi SA, Saddiwal RS, Nisa SU (2017) Age estimation in Indian adults by the coronal pulp cavity index. J Forensic Dent Sci 9:177. 10.4103/jfo.jfds_60_16 29657499 10.4103/jfo.jfds_60_16 PMC5887645

